# Balint groups: an effective tool for improving health professionals’ perceived well-being

**DOI:** 10.1186/s13584-024-00618-8

**Published:** 2024-08-01

**Authors:** Ruth Kannai, Shai Krontal, Tamar Freud, Aya Biderman

**Affiliations:** 1 Siaal Research Center for Family Medicine and Primary Care, Department of Family Medicine, The Haim Doron Division of Community Health, Faculty of Health Sciences, Ben-Gurion University of the Negev, POB 653, Beer-Sheva, 8410501 Israel; 2 Clalit Health Services, Jerusalem district, Jerusalem, Israel; 3 Department of Family Medicine, Maccabi Healthcare Services, Tel Aviv, Israel; 4 Department of Family Medicine, Faculty of Medical & Health Sciences, Tel Aviv University, Tel Aviv, Israel; 5 Research Unit, Clalit Health Services, South District, Beer-Sheva, Israel

**Keywords:** Balint groups, Physician well-being, Burnout, Self-care, Professional identity, Patient care

## Abstract

**Background:**

Physician burnout is a common problem that negatively impacts their well-being and patient care. Balint groups (BGs) deal with doctor-patient relationships. Previous studies that have demonstrated the positive effects of BGs are descriptive and based on small sample sizes. This study aims to evaluate the perceptions of health professionals who participated in BGs, determine the impact of BGs on their personal and professional well-being, and identify the factors related to these positive outcomes.

**Methods:**

On January and February 2023 the authors have distributed a questionnaire to 142 healthcare providers in a conference and internet networks. Most respondents were family physicians.

**Results:**

Participation in BGs is seen to have a positive impact on healthcare professionals’ perceived well-being and professional development. Respondents who had participated in the BG reported a reduction in burnout, increased empathy, and enhanced professional identity and relationships with patients and colleagues. The study also highlighted the importance of duration of participation in BG, with attendance longer than 5 years linked to significantly more positive outcomes compared to less than 1 year. In a logistic regression analysis two factors were significantly associated with self-reported well-being: attending BGs for more than five years and perceiving BGs as a means of relieving burnout.

**Conclusions:**

The findings suggest that medical organizations should encourage the regular availability of BGs to support physicians’ well-being.

**Supplementary Information:**

The online version contains supplementary material available at 10.1186/s13584-024-00618-8.

## Background

The phenomenon of professional burnout has been observed across various professions and is characterized by emotional exhaustion, depersonalization, and a sense of low achievement [1]. Among physicians, it is very common, with up to 80.5% prevalence of doctors experiencing symptoms of burnout [2].

Professional burnout can negatively impact the health and quality of life of physicians, causing emotional and physical exhaustion, fatigue, lack of energy, cognitive difficulties, and problems with concentration. These factors may affect a doctor’s ability to make sound decisions, and it is not surprising that physicians’ burnout has been linked to decreased productivity, harm to patients and a decline in the quality of treatment [3], Specifically, physician burnout can result in reduced patient satisfaction, lower adherence to treatment, an increase in medical errors, and a decrease in empathy [4, 5]. During the residency period, it was found that burnout levels were at their highest and feelings of overall health were at their lowest, as indicated by factors such as physical activity, sleep patterns, substance use, and general health perception [6].

The 2021 survey on burnout levels in the Israeli health system [7], included 34,000 health system workers, showed an average burnout level of 3.4 on the Maslach Burnout Inventory - HSS, which is higher than the 2.2 recorded in a survey of 20,000 workers from other professions. Furthermore, 34% of the surveyed employees experienced burnout at level 4 or higher. Physicians had the highest burnout average of 3.6, followed by nurses at 3.4. Residents were the most worn-out doctors, with a burnout level of 4.4.

In a cross-sectional study completed by 90 family medicine residents throughout Israel [8], the rate of clinically significant burnout was 14.4%. A strong association was shown between burnout and psychological characteristics. The study concluded that the relatively low prevalence of burnout among family medicine residents could be attributed to the integration of burnout prevention programs into academic courses during their residency.Despite numerous studies indicating the prevalence of professional burnout among residents, there are only few controlled studies on intervention programs aimed at preventing burnout and promoting personal health and well-being among this group. The existing studies primarily focus on mindfulness-based observation techniques or cognitive-behavioral methods to alleviate stress [9–11]. The effectiveness of various burnout prevention interventions has been tested in multiple medical schools and residency programs. Some examples include reflective writing and peer support [12].

These methods share a common goal of increasing self-awareness, supporting personal resilience, and fostering mutual support. Research has shown that such interventions are effective in preventing burnout, promoting professional resilience, and cultivating a sense of well-being for therapists [13]. In addition to these interventions, improving working conditions, minimizing bureaucratic tasks, and enhancing the doctor’s work environment have also been proven to prevent and reduce burnout [14].

The family medicine residency program at Ben-Gurion University of the Negev provides several courses aimed at preventing burnout. These include narrative and reflective writing, mindfulness, and yoga/relaxation. Additionally, all residents participate in two semesters of monthly BG sessions during their four-year residency. Most participants in these courses have provided positive feedback, expressing high satisfaction with the experience [15].

In the 1950s, the psychoanalysts Michael and Enid Balint established BGs to assist healthcare professionals in gaining a deeper understanding of the emotional aspects of the doctor-patient relationship [16]. The ultimate goal of these groups is to enhance the therapeutic potential of healthcare providers [17]. BGs were originally created for physicians to discuss their experiences with patients and provide emotional support to one another. However, the model has been adapted for other healthcare providers, including nurses, social workers, and mental health professionals. BGs typically meet regularly to discuss cases regarding doctor-patient relationships. The groups are led by two trained leaders, who encourage reflection and discussion of the emotional aspects of patient care.

An Israeli article provides a descriptive account of how participation in BGs helps to establish and maintain a network of professional support among healthcare professionals [18]. Another Israeli article, describes a BG at Soroka Hospital in the Negev [19] and claims that these groups prevent burnout and improve doctor-patient relationships. In a cross-sectional study in Serbia, doctors who participated in BGs, showed a reduction in burnout indices, compared to doctors who did not participate [20]. Three articles [21–23] that utilized the Psychological Medical Inventory (PMI) and relied on self-reporting by a small group of nurses and interns found that participation in BGs led to an improvement in the psychological self-efficacy of the participants. One of the studies [20] also showed a decrease in burnout indices after 10 months of participating in a BG, but not after 6 months. A German Balint Group Questionnaire (BGQ-G) examines process variables that contribute to a favorable outcome and positive impact of Balint work [24]. A study focused on validating and establishing the reliability of the BGQ-G in China [25]. mainly addressed the learning of transference dynamics, emotional and cognitive learning, and case mirroring dynamics within the group. However these studies did not included questions related to well-being and burnout prevention in their questionnaires. A descriptive qualitative research of nine participants found that BGs were effective in increasing job satisfaction and preventing burnout among general practitioners [26]. In a Chinese study, a group of 36 first-year residents were randomly assigned to either 6 months of BG participation (the study group) or a waiting group. The study group showed an improvement in burnout indices after the intervention [27].

BGs are recognized and actively operating in most family medicine departments in Israel. In recent years, there has been a growth in the number of BGs designed for physicians from other professions and other healthcare workers. The Balint community in Israel is developing and collaborating with the international Balint society [28].

In this study, we aimed to assess the perception of a large group of health professionals who have been participating in BGs, regarding the impact on their perceived personal and professional well-being, and to describe the factors related to these positive outcomes.

The research had four main objectives:

1. To examine the relationship between participation in BGs and self-perception of well-being, self-care, creating a peer group, patient care, and resilience among family doctors and other health workers in Israel.
2. To investigate the impact of the duration of participation in BGs and the obligation to participate (yes/no) on the participant’s reported sense of well-being, both personally and professionally.
3. To identify barriers to participation in BGs among family doctors who have never participated in such a group.
4. To explore whether the participants sense of resilience and personal well-being is related to variables such as the participant’s age, gender, origin and religion, voluntary or mandatory participation in the group, the stage and seniority of their career, length of participation in BGs and their specific profession.

## Methods

A cross-sectional study that included:

1. Family doctors in Israel who are part of the “Kehiladocs” internet network, a self-managed network of residents and specialists in family medicine consisting of approximately 440 participants.
2. The mailing list of current and former participants in the Balint conferences in Israel, which are held twice a year. The list is managed by one of the authors (S.K.) and includes family physicians, social workers, psychologists, physicians from other specialties, nurses and psychiatrists. This list has around 150 participants.
3. Participants of the Heimar (Israeli Society of Family Medicine Teachers) conference held in February 2023, which had approximately 100 participants.

The questionnaire (Additional file 1) was distributed twice to the family doctors in the “Kehiladocs” network from January 2023, and to the mailing list of the Balint circle in Israel in February 2023. Additionally, participants of the “Heimar” conference (February 2023) were asked to complete the questionnaire using a link distributed at the conference.

In this analysis, we included people who belong to the kehilaDocs email network and who answered all or part of a questionnaire distributed twice by email on this list.

Nearly all respondents who participated in the “Heimar” conference and/or are members of Balint mailing list are also registered in the KehilaDocs email group. Therefore, we assume that the 142 respondents, most of whom were family physicians, were members of the Kehiladocs group.

### Statistical analysis

Statistical analyses were conducted using SPSS software version 29.0 (IBM, Armonk, NY). Categorical variables, such as sex, were presented as frequencies and percentages, while continuous variables, such as age, were presented as means and standard deviations.

We compared attitudes toward participation in BGs and self-perception of well-being based on the following factors: duration of participation in BGs (less than one year/1–4 years/5 years and above), obligation to participate in BG (yes/no), engagement in management (yes/no), engagement in teaching (yes/no), and involvement in activities that contribute to the prevention of burnout (yes/no). Chi-square tests were used to compare categorical variables. For continuous variables, since the responses were not normally distributed, we used non parametric test: Kruskal-Wallis test for 3 group comparisons and Mann-Whiteny U test for 2 group comparisons.

Logistic regression analysis was performed to determine factors related to high self-perception of professional well-being. The model included: socio-demographic variables, length of participation in BGs and two attitude questions: “Participation in the group has improved my relationships with patients” and “Relieves my burnout”. Statistical significance was set at *P* < 0.05.

The study received approval from the Human subject research committee at the Faculty of Health Sciences, Ben-Gurion University of the Negev, Beer-Sheva, Israel (approval No. 51-2022 at January 2023). All the participants agreed to participate in the study.

## Results

### Study population characteristics

Altogether, 142 health care providers filled out the questionnaire. The characteristics of the study population are presented in Table 1. The mean age was 51.07 (SD = 10.3), 103 females (75.2%), 113 (81.3%) were born in Israel, 93 (66.9%) defined themselves as secular Jews, 38 (27.3%) as religious or traditional Jews. The majority of respondents were board certified family physicians (*N* = 117, 84.8%), 115 (85.8%) completed their academic studies in Israel. Most (*N* = 64, 46%) of the respondents had a professional experience of more than 20 years. An impressive number of respondents were involved in teaching(*N* = 115, 82.7%), and over 50% were engaged in management at some level (*N* = 70, 50.4%). Regarding self-care, 105 (76.1%) took some action to prevent burnout, with 91 (86.7%) engaging in physical activity, 49 (46.7%) have hobbies, 20 (19%) practice yoga, and 14 (13.3%) practice mindfulness.

**Table 1:** Characteristics of the study population (*N* = 142)

	*N*	%
**Age**		
Average ± SD	51.07 ± 10.3	
Median	50	
Range	34–84	
	137	(Mis = 5)
**Gender**		
Male	34	24.8%
Female	103	75.2%
	137	(Mis = 5)
**Country of birth**		
Israel	113	81.3%
Other	26	18.7%
	139	(Mis = 3)
**Religion**		
Secular Jewish	93	66.9%
Religious / traditional Jewish	38	27.3%
Moslem	3	2.1%
Other	5	3.6%
	139	(Mis = 3)
**Profession**		
Family medicine resident	5	3.6%
Family medicine specialist	117	84.8%
Psychologist	3	2.2%
Social worker	3	2.2%
Occupational Therapist	6	4.3%
Other	4	2.9%
	138	(Mis = 4)
**Country of academic studies**		
Israel	115	85.8%
Other	19	14.2%
	134	(Mis = 8)
**Years of professional practice**		
Up to 5 years	9	6.5%
6–10 years	29	20.9%
11–19 years	37	26.6%
More than 20 years	64	46.0%
	139	(Mis = 3)
**Management position**		
No	69	49.6%
Yes	70	50.4%
	139	(Mis = 3)
**Teaching**		
No	24	17.3%
Yes	115	82.7%
	139	(Mis = 3)
**Participation in any burnout prevention activity**		
No	33	23.9%
Yes	105	76.1%
	138	(Mis = 4)
**What activity?**		
Physical Exercises	91	86.7%
Hobbies	49	46.7%
Yoga	20	19.0%
Mindfulness	14	13.3%
Other	26	24.8%
	105	

### Characteristics of participation in Balint groups

Table 2 shows the respondents’ experience with Balint groups: 54 (38.0%) are currently participating in the BG, while 67 (47.2%) have participated in the past, and 21 (14.8%) have never participated. Among those who participated, 38 (32.5%) did so for less than a year, 38 (32.5%) for 1–4 years, 21 (17.9%) for 5–9 years, and 20 (17.1%) for more than 10 years.

**Table 2:** Characteristics of participation in BGs (*N* = 142)

	*N*	%
**Participation in Balint groups**		
Current participation	54	38.0%
Participation in the past	67	47.2%
Never participated	21	14.8%
	142	
**Length of participation in Balint Groups?**		
Less than one year	38	32.5%
1–4 years	38	32.5%
5–9 years	21	17.9%
More than 10 years	20	17.1%
	117	(Mis = 4)
**Periods in your professional career in which you participated in a Balint group (more than one possible answer)**		
**During medical School**	9	6.0%
Obligatory	5	55.6%
Mandatory	4	44.4%
	9	
**In my residency program**	79	52.7%
Obligatory	12	15.2%
Mandatory	38	48.1%
	50	(Mis = 29)
**As a medical specialist**	79	52.7%
Obligatory	49	94.2%
Mandatory	3	5.8%
	52	(Mis = 28)
**As a balint group facilitator**	31	20.7%
Obligatory	17	89.5%
Mandatory	2	1.4%
	19	(Mis = 12)
	121	

Most of the respondents participated in the BG as Family Medicine residents (*N* = 79, 52.7%), and medical specialists (*N* = 79, 52.7%). Only a minority of respondents (*N* = 9, 6.0%) participated in the BG during their under graduate education as medical students. 31 (20.7%) of the respondents lead a BG currently.

### Attitudes regarding participation in BGs

**Fig. 1:**
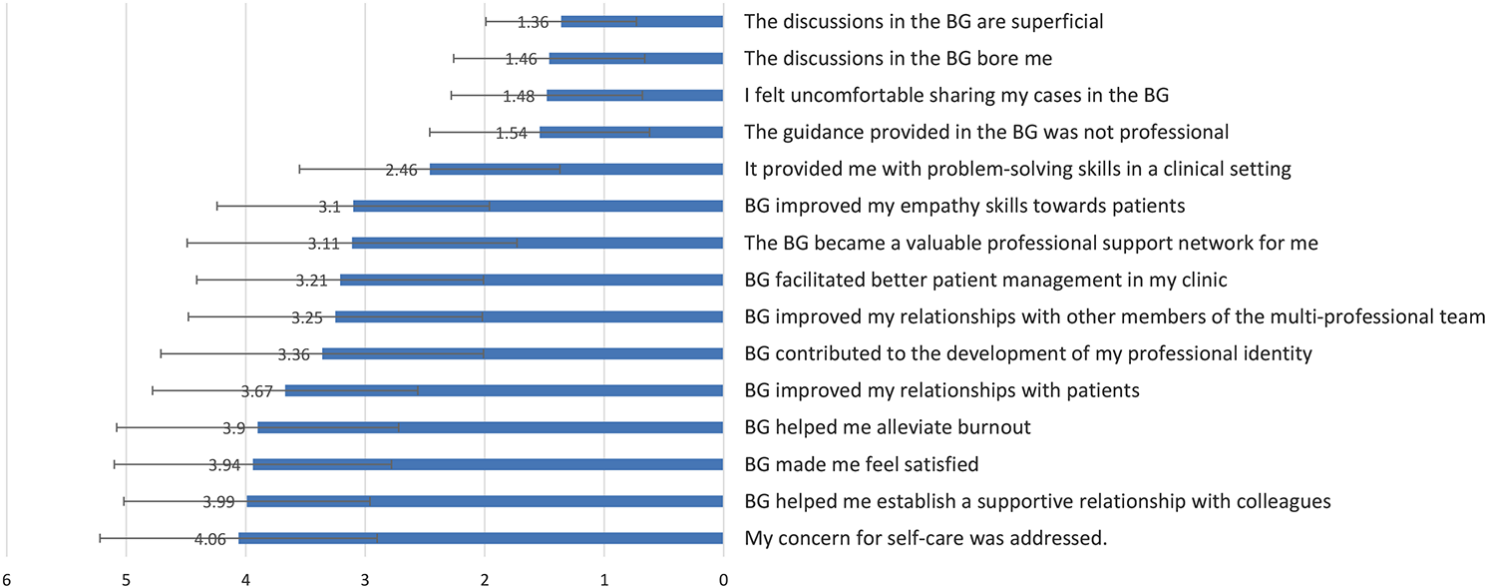
Level of agreement with the statements concerning participation in the last BG* (*N* = 121). *Scale of 1-5, 1 indicates complete disagreement and 5 indicates substantial agreement; BG-Balint group

The level of agreement with statements concerning participation in the last Balint Group was rated from 1 = do not agree at all, to 5 = agree to a large extent (Fig. 1). The highest 3 scores were given to: “My concern for self-care was addressed” 4.06 (1.16), “helped me establish a supportive relationship with colleagues” 3.99 (1.03), and “made me feel satisfied” 3.94 (1.16). Low scores were given to negative statements regarding the BG: “I felt uncomfortable sharing my cases in the BG” 1.48 (0.8), “the discussions in the BG bore me” 1.46 (0.8) and “the discussions in the BG are superficial” 1.36 (0.63).

56.7% of the participants (*N* = 68) answered that participation in BG is important/very important, for their professional well-being and 22.5% (*N* = 27) answered that participation is not important or not important at all (mean score 2.28(1.29)).

Among 21 respondents who have never participated in a BG, the reasons for non-participation were mainly because it was offered at the expense of private or family time (52.4%, *N* = 11), or because there is no BG in their area of residence, or it is not accessible to them (42.9%, *N* = 9). Only 9.5% (*N* = 2) do not believe in the usefulness of the group, and 4.8% (*N* = 1) did not trust the group.

There were no statistically significant differences in attitudes regarding participation in BGs between: respondents who participate in other activities to prevent burnout compared to those who do not participate; between secular Jews and religious or traditional Jews and between those for whom participation in the BG was mandatory and those who participated in the group by choice.

### Lengths of participation in BGs

Table 3 describes differences in attitudes regarding participation in BGs according to duration of participation in the BGs: less than a year (*N* = 38), 1–4 years (*N* = 38), 5 years and more (*N* = 41).

**Table 3:** differences in attitudes regarding participation in BGs according to duration of participation in the BGs

	Length of participation in Balint Groups												
	Less than a year			1–4 years			5 years or more			*p* ^1^	*p* ^2^	*p* ^3^	*p* ^4^
	Mean	SD	*N*	Mean	SD	*N*	Mean	SD	*N*				
**Level of agreement with the statements concerning participation in the last BG**													
My concern for self-care was addressed	3.61	1.386	38	4.08	1.124	38	4.4	0.841	40	0.01	0.214	0.008	0.642
BG helped me establish a supportive relationship with colleagues	3.45	1.155	38	4.03	0.915	38	4.43	0.813	40	< 0.001	0.031	< 0.001	0.216
BG made me feel satisfied	3.29	1.374	38	4.03	1.102	38	4.45	0.639	40	< 0.001	0.01	< 0.001	0.254
BG helped me alleviate burnout	3.37	1.344	38	3.84	1.189	38	4.37	0.807	40	< 0.001	0.17	< 0.001	0.151
BG improved my relationships with patients	3	1.185	38	3.68	0.962	38	4.22	0.832	40	< 0.001	0.011	< 0.001	0.056
BG contributed to the development of my professional identity	2.47	1.33	38	3.63	1.239	38	3.93	1.095	40	< 0.001	< 0.001	< 0.001	0.876
BG improved my relationships with other members of the multi-professional team	2.55	1.155	38	3.42	1.13	38	3.69	1.104	39	< 0.001	0.003	< 0.001	0.883
BG facilitated better patient management in my clinic	2.61	1.178	38	3.53	1.059	38	3.43	1.174	40	< 0.001	0.002	0.006	1
The BG became a valuable professional support network for me	2.45	1.329	38	3.39	1.152	38	3.5	1.45	40	< 0.001	0.007	0.002	1
BG improved my empathy skills towards patients	3.24	1.24	38	3.87	1.143	38	4.25	0.84	40	< 0.001	0.037	< 0.001	0.396
It provided me with problem-solving skills in a clinical setting	2.13	1.018	38	2.53	0.979	38	2.62	1.161	39	0.108	0.319	0.141	1
The guidance provided in the BG was not professional	1.5	0.862	38	1.81	1.126	37	1.35	0.736	40	0.087	0.436	1	0.089
I felt uncomfortable sharing my cases in the BG	1.82	0.982	38	1.5	0.797	38	1.15	0.427	40	< 0.001	0.225	< 0.001	0.138
The discussions in the BG bore me	1.82	1.036	38	1.38	0.721	37	1.25	0.494	40	0.005	0.05	0.005	1
The discussions in the BG are superficial	1.61	0.855	38	1.32	0.53	37	1.2	0.405	40	0.016	0.161	0.015	1
How important is participation in BG for your professional well-being? **	3	1.185	38	2.18	1.27	38	1.68	1.023	40	< 0.001	0.008	< 0.001	0.166

*Scale of 1–5, 1 indicates complete disagreement and 5 indicates substantial agreement; ** Scale of 1–5, 1 = very important, 5 = not important at all

*Abbreviation* SD- Standard deviation; BG-Balint group; P^1^ = difference between the three groups; P^2^ = difference between less than a year and 1–4 years;

P^3^ = difference between less than a year and 5 years or more; P^4^ = difference between 1–4 years and 5 years or more

The longer the respondents participated in the BG, the higher the scores were for all positive statements, with statistical significance. In all positive statements there was a significant difference (P value < 0.001) between the three groups according to the duration of participation, less than a year, 1–4 years, and 5 years or more. These differences were markedly significant between less than a year of participation in the BG and 5 years or more. There were no statistically significant differences between 1–4 years of participation in the BG and 5 years or more.

In response to a direct question, regarding the importance of participation in a BG for professional well-being (1 = very important, 5 = not important at all) the group of participants of more than 5 years scored 1.68 compared to a score of 3 for people who participated in the BG for less than a year.

Participants with less than a year of experience were more likely to report feeling uncomfortable sharing their cases, while those who had been in the group for a longer period, particularly for more than five years, were more comfortable sharing(*p* < 0.0001).

### Factors related to self-perception of professional well-being

A logistic regression analysis was performed to determine factors related to self-perception of professional well-being (Table 4). The results showed that only two factors were significantly associated with perceived well-being: attending BGs for more than five years (OR = 7.463, 95% CI 1.573–35.393) and perceiving BGs as a means of relieving burnout (OR = 3.070, 95% CI 1.505–6.263). Other factors, including age, gender, professional experience, involvement in teaching or management, and currently participating in a BG, were not related to perceived well-being.

**Table 4:** Logistic regression analysis to determine factors related to self-perception of professional well-being

Variable	OR	*P* value	95%CI
Age	0.996	0.896	0.932–1.063
Gender	2.259	0.187	0.673–7.584
Management position	0.575	0.276	0.213–1.556
Teaching	3.613	0.079	0.861–15.162
Seniority in the profession	0.509	0.331	0.131–1.985
*Length of participation in BGs*:			
Less than a year	Reference		
1–4 years	3.412	0.060	0.950-12.257
5 + years	7.463	0.011	1.573–35.393
BG improved my relationships with patients	1.054	0.871	0.549–2.022
BG helped me alleviate burnout	3.070	0.002	1.505–6.263
Current participation in a BG	1.090	0.871	0.384–3.092

*Abbreviation* BG-Balint group; -2LL = 109.201; Hosmer & Lemeshow = 0.928

## Discussion

BGs are widespread in Israel and participation is available to both medical students and healthcare professionals, including residents and board-certified family physicians. Some medical schools and residency programs require at least one semester of mandatory participation in BGs during their clinical studies, while most groups are optional. Various organizations such as the Balint Association in Israel, health care organizations, hospitals, residency programs and universities offer BGs. A minority of groups are private, and participants pay fees to the group leaders. Despite the potential benefits of BGs, research on their effectiveness is limited and often lacks rigorous methodology. There are only a few studies published on the outcomes of BGs in general, and even fewer studies focus specifically on the impact of BGs on preventing burnout [29]. In one qualitative study [30], a focus group comprising 19 BG participants revealed several main themes. These included investigating emotions, the development of the physician’s identity, as well as safety within the group and with the leader. Participants reported experiencing relief from stress and an enhanced ability to understand the emotional aspect of patient encounters. This theme could be linked to well-being. Most of these studies are descriptive and limited in scope. One possible reason for the lack of studies is the difficulty of isolating the impact of the BG from other factors that influence medical education for students and residents, as well as the various working environments of physicians. In fact, it is challenging to unequivocally isolate participation in the BG as a factor in preventing burnout and developing resilience.

The uniqueness of our research lies in the large sample size; A total of 142 participants. Of note, 21 (14.8%) respondents who had never participated in the BG also provided insights into the barriers to joining a BG.

Most of the respondents were women. It can be assumed that this is related to the high proportion of women among the health care providers in Israel and the high proportion of women who participate or lead BGs in particular.

Almost half (*N* = 80, 50.4%) of the respondents are involved in management positions, while over 80% (*N* = 115) are engaged in teaching. This particular group holds significant experience and has a considerable influence on the medical system and on medical education, especially in residency programs. Therefore, their positive experience in participating in BGs could potentially impact the integration of BGs into the medical system and at all levels of medical education.

The results of this study provide evidence for the positive effects of BG participation on healthcare professionals’ well-being and professional development. The majority of respondents indicated that participation in the BG was important to their professional well-being and improvement in various aspects of their professional and personal lives. These findings are consistent with previous research indicating that BG participation can reduce burnout, increase empathy and communication skills, and enhance professional identity and relationships with patients and colleagues [26].

We found that the duration of participation in the BG is an important factor in the positive outcomes reported by the respondents. The longer the duration of participation, the higher the scores for positive statements such as improved relationships with patients, colleagues, and multi-professional teams, as well as reduced burnout and increased professional identity. Participants who have been attending the group for less than one year and those who have attended for a longer period show a notable difference in their comfort level when it comes to sharing cases within the group. The statistical significance of these findings is most pronounced between individuals who have participated for less than one year compared to those who have been attending for more than five years. In the regression analysis, we found two factors related to the respondent’s sense of professional well-being: Attendance in BGs for more than 5 years and the perception that it relieves their burnout.

A possible explanation for this finding is that there is a combination of “cause” and “effect”: not only that the BG has a positive impact on the attendants, but also that those who experience theses positive effects stay on and continue to attend BGs for long periods of time. A similar connection can exist between the increase in well-being and the perceived reduction in burnout.

The study also highlights some of the barriers to participation in BG, such as conflicts with private or family time and lack of availability in certain geographical areas. Addressing these barriers may be important for increasing participation rates and extending the benefits of BGs to more healthcare professionals.

Based on these findings, we assert that the responsibility for establishing BGs lies with employers, such as the state, HMO’s, hospitals, and professional unions. Similar to other professional training, BGs should take place during work hours and at the workplace. The Balint Association in Israel is currently working towards establishing more groups in hospital departments and in large clinics for selected professional groups or for multi professional teams. Additionally, most BGs are now included in the curriculum in family medicine residency departments. We suggest that this trend should continue and expand to offer health professionals the option to participate in these groups at their workplace during work hours.

One limitation is our study’s cross-sectional design, which makes it challenging to isolate the impact of the BG from other influencing factors in medical education and physicians’ working environments.The study did not find significant differences in BG participation based on religious affiliation or whether participation was mandatory or voluntary. This suggests that BGs may be effective across diverse groups of healthcare professionals and organizational contexts.

We expected to find greater benefit and more positive outcomes from voluntary participation in the BG rather than a mandatory one. Given the nature of BG, it requires trust, openness, and participants’ willingness to share their conflicts within the doctor-patient relationships in a supportive and confidential setting. However, the sample size in our study was not sufficient to validate any differences between voluntary and mandatory participation. Qualitative research with groups of both types could provide a more precise answer to this question.

Question 4 of our survey pertains to the participant’s most recent BG experience. Given the diverse range of responses, some participants may have attended a session recently, while others may recall experiences from a more distant past. This discrepancy introduces a potential recall bias, complicating comparisons across the participant groups identified in Question 2. However, In the multivariate analysis, participation, whether current or past, was not found to have an impact on perceived well-being.

We also observed that there were fewer participants from minority or immigrant backgrounds in BGs. This may be related to different cultural or educational backgrounds, where it is less acceptable to share or even to express feelings and difficulties among colleagues. In our study, the representation of these groups was lower than their proportion in the population, highlighting the need for further investigation into this issue, too.

The low response rate of 32% (142 out of 440 questionnaires sent) is typical for email based surveys of physicians. Factors such as time constraints, survey length, and perceived relevance may have contributed. Non-response bias could skew results, as respondents may represent a subset of physicians more inclined towards research or with specific interest in BG.

This study was conducted in a specific geographic area and healthcare system, and further research is needed to determine the generalizability of these findings to other contexts.

Another limitation of this study is its reliance on self-report measures, which may be subject to biases and inaccuracies, such as “social desirability bias”. Future research could incorporate more objective measures of outcomes such as patient satisfaction or clinical outcomes. Further research in these areas could help to refine and optimize the BG intervention for its maximal effectiveness.

## Conclusions

This study adds to the growing body of research supporting the use of BGs as a tool for promoting healthcare professionals’ well-being and professional development. Healthcare organizations may benefit from considering the implementation of BGs as part of a comprehensive approach to preventing and addressing burnout and promoting a culture of reflective practice and professional growth.

It is worth noting that for those who did not participate in BGs the primary barrier is the availability and accessibility of such a group rather than criticism of the process itself.

There is a gap between the acknowledged effectiveness of Balint for experienced Balint participants on their professional lives and the recognition of Balint by healthcare organizations as a valuable and significant tool, particularly in a time when the workload on healthcare workers is so immense and burnout rates are high and especially in the post-Covid era.

Enhancing the professional well-being of employees is desirable. The establishment of a Balint system for healthcare professionals in healthcare organizations is a collaborative effort involving various officials such as management, financial staff, the training system, health professionals, and Balint leaders. To launch and maintain such a program, it is essential to have visionaries who can lead the effort. Achieving this important goal requires collaboration from all professionals involved, as the saying goes, ‘It takes a village’ [31].

### Electronic supplementary material

Below is the link to the electronic supplementary material.

Supplementary Material 1

## Data Availability

The datasets used and/or analysed during the current study are available from the corresponding author on reasonable request.

## Abbreviations

BGs: Balint groups
HSS: Maslach Burnout Inventory
