# Supplementary material for: Balint groups: an effective tool for improving health professionals’ perceived well-being
Source: Isr J Health Policy Res. 2024 Aug 1;13:31. doi: 10.1186/s13584-024-00618-8 (PMC11293207; doi:10.1186/s13584-024-00618-8)
Supplement: Supplementary file 1 — Supplementary Material 1 [file 13584_2024_618_MOESM1_ESM.docx]

**Additional file 1: Questionnaire**

**1. Have you ever participated in a Balint group, either currently or in the past?**

A. I am currently participating in a Balint group.

B. I participated in a Balint group in the past.

C. I have never participated in a Balint group.

**2. How long have you been involved in Balint groups?**

A. Less than a year.

B. 1-4 years.

C. 5-10 years.

D. More than 10 years.

**3. Please indicate during which periods of your professional career you have participated in the Balint Group (BG). (you can answer more than one answer)**

| **Period** | **Participation in BG** | | **Type of group** | |
| --- | --- | --- | --- | --- |
| **A. During my under graduate studies** | Yes | No | Mandatory | Obligatory |
| **B. During Residency** | Yes | No | Mandatory | Obligatory |
| **C. As a specialist** | Yes | No | Mandatory | Obligatory |
| **D. As a group leader** | Yes | No | Mandatory | Obligatory |

**4. Please mark your degree of agreement with the following sentences regarding the last Balint group (BG) you participated in: where: 1= do not agree at all 5= agree to a large extent.**

|  |  | **1= Do not agree at all** | **2** | **3** | **4** | **5= Agree to a large extent** |
| --- | --- | --- | --- | --- | --- | --- |
| **1** | BG improved my relationships with patients |  |  |  |  |  |
| **2** | BG helped me establish a supportive relationship with colleagues |  |  |  |  |  |
| **3** | BG improved my relationships with other members of the multi-professional team |  |  |  |  |  |
| **4** | BG provided me with problem-solving skills in a clinical setting |  |  |  |  |  |
| **5** | BG helped me alleviate burnout |  |  |  |  |  |
| **6** | BG facilitated better patient management in my clinic |  |  |  |  |  |
| **7** | BG improved my empathy skills towards patients |  |  |  |  |  |
| **8** | BG made me feel satisfied |  |  |  |  |  |
| **9** | My concern for self-care was addressed in BG. |  |  |  |  |  |
| **10** | The BG became a valuable professional support network for me |  |  |  |  |  |
| **11** | BG contributed to the development of my professional identity |  |  |  |  |  |
| **12** | The discussions in the BG bore me |  |  |  |  |  |
| **13** | the discussions in the BG are superficial |  |  |  |  |  |
| **14** | I felt uncomfortable sharing my cases in the BG |  |  |  |  |  |
| **15** | The guidance provided in the BG was not professional |  |  |  |  |  |

**5. How important is participation in the Balint group for your professional well being?**

**1=** Very important **2 3 4 5** = Not important at all

**6. If you have never participated in a Balint group, please indicate the reason(s) why. (You may select more than one answer.)**

A. There is no Balint Group in my area or it is not accessible to me.

B. I was not offered the opportunity to participate.

c. I do not believe that participating in a group would be beneficial to me.

D. I think that participating in the Balint Group could be harmful to me.

E. I did not trust the other participants in the BG.

F. I did not trust the moderator of the BG.

G. I did not want to lose my private or family time for the BG**.**

H. Other reasons:__________________________________

1. **Do you currently engage in any activities to prevent burnout at work? Please select** : Yes No

**If Yes, please specify which activities you engage in:**

A. Exercises

b. Hobbies

C. Yoga

D. Mindfulness

C. Other: _________________________

**9. Age**

**10. Gender:**

A. Female

B. Male

C. Other/prefer not to answer

**11. Country of birth**

A. Israel

B. Other

**12. How do you define yourself religiously?**

A. A secular Jew

B. A religious or traditional Jew

C. Moslem

d. Christian

G. Other____________________________

**13. What is your Profession?**

A. Medical resident

B. Medical Senior

C. Psychologist

D. Social worker

E. Occupational Therapist

F. Other_______________________________

**14. Country of academic studies**

A. Israel

B. Other

**15. Years of professional practice**

A. Up to 5 years

B. 6-10 years

C. 11-19 tears

D. More than 20 years

**16. Are you engaged in a management position ?**

A. Yes

B. No

**17. Are you engaged in teaching ?**

A. Yes

B. No
